# 575 Disparities in Burn Hospitalizations in New York City 2010-2019

**DOI:** 10.1093/jbcr/irae036.209

**Published:** 2024-04-17

**Authors:** Christine Lopez, Diego Luna, Perry Sheffield

**Affiliations:** Icahn School of Medicine at Mt. Sinai, New York, NY; Johns Hopkins University Applied Physics Laboratory, New York, NY; Icahn School of Medicine at Mt. Sinai, New York, NY; Johns Hopkins University Applied Physics Laboratory, New York, NY; Icahn School of Medicine at Mt. Sinai, New York, NY; Johns Hopkins University Applied Physics Laboratory, New York, NY

## Abstract

**Introduction:**

Burn injury is a major source of morbidity, mortality and economic cost in the United States. Current literature does not describe the epidemiology of burn injury in New York City within the last decade. This analysis establishes an important understanding of the recent burden and demographics of burn injury and serves to support tracking of the effectiveness of ongoing policies that aspire to reduce the incidence and disparities within the realm of burn injury. By looking at injury rates by borough and race/ethnicity, we identify at-risk populations to support targeted public health interventions.

**Methods:**

This analysis used data from the de-identified NYS Department of Health Statewide Planning and Research Cooperative System (SPARCS) publically available online database of inpatient records from 2010-2019. Incidence rates were calculated using the 2010 Decennial Census data and boroughs were compared using Zip Code Tabulation Areas (ZCTA). All data was analyzed using Python.

**Results:**

Data included 11,444 burn injury hospitalizations from 2010-2019 across NYC for all ages. Data reviewed over the 10 years showed some persistent and shifting distribution patterns across geography and demographics. Staten Island had the highest incidence every year while Brooklyn and Queens had the highest total number of burn injuries compared to the other boroughs. Males had a higher rate of burn injury than females every year in NYC from 2010-2019. Age 0-17 had the highest burn injury rate in every year except 2016, which had 70+ as the highest rate. 70+ had the second highest rate of burn injury in all other years. In the 5 boroughs those who identified as white had the highest rate of burns in 2010 and 2011. This data shifted over time, with incidence rates in the Black/African American, Multi-Racial, and Other Race category increasing. Average length of stay in New York City for inpatient burn hospitalization was 8.12 days from 2010-2019 excluding patient stays that were >120 days.

**Conclusions:**

This New York City data analysis provides a unique insight into the prevalence and disparities of burn injuries in an urban health setting. Data augments existing literature regarding differences in burn injury and can guide future injury prevention initiatives.

**Applicability of Research to Practice:**

This descriptive analysis provides a basis by which work can be done across New York state that focuses on preventative public health measures to reduce burn injury. The work also creates further research questions on why specific areas or populations are more impacted by burn injury.

